# Deep Learning Scoring Model in the Evaluation of Oral English Teaching

**DOI:** 10.1155/2022/6931796

**Published:** 2022-07-30

**Authors:** Yamei Liu, RongQin Li

**Affiliations:** ^1^School of Foreign Languages, Shenyang Institute of Engineering, Shenyang 110136, Liaoning, China; ^2^School of Artificial Intelligence and Big Data, He University, Shenyang 110163, Liaoning, China

## Abstract

This study is aimed at improving the accuracy of oral English recognition and proposing evaluation measures with better performance. This work is based on related theories such as deep learning, speech recognition, and oral English practice. As the literature summarized, the recurrent neural network was the calculation standard, and the oral English speech recognition indicators were the main basis on which an English speech recognition model was constructed. Then, 20 English majors and 5 sets of English sentence patterns were randomly selected as the research objects. The correction standards for English oral errors were introduced into the model to achieve further improvement. The research results showed that the average concordance rate of speech recognition reached 91% through the model test. The concordance rates of words, speech, and intonation in recognition were 89%, 91%, and 86%, respectively. The model could be used as an evaluation system for English speech recognition. Therefore, the application of the deep learning scoring model in the evaluation of oral English teaching was researched in this work, which provided an effective basis for the evaluation of intelligent English teaching.

## 1. Introduction

Since the beginning of the 21st century, global economic exchanges have continued to deepen, and there have been more and more opportunities for people from all countries to communicate with each other. A single language can no longer meet the needs of communication [1]. English, as one of the most widely used languages in the world, has gradually increased its proportion in teaching in China. Vocabulary, grammar, and oral English are the most important factors for improving the level and capability of English education [2]. However, due to the increasingly prominent issues such as the limited number of English teachers and the lack of English learning resources, a measure that can identify, evaluate, and correct English grammar is urgently needed [3]. Compared with manual evaluation methods, new network technology is a feasible method to evaluate oral English [4].

Fandiño et al. [5] believe that the development of the ability to speak English needs to be at the corresponding education level, and the motivation to learn a foreign language is a complex process that is strongly affected by many external factors. Khasawneh [6] believes that various factors like speaking, reading, writing, and listening are certain difficulties encountered in English learning, so it is necessary to strengthen the cultivation of primary English learning ability. Chrupała [7] finds that when students learn a language, they need to rely on different emotions and organs, of which the most important are the visual pattern signals that occur simultaneously with oral language. Since English is a language in daily communication, oral practice must be strengthened, not just stop at the learning of visual simulation. Therefore, Barrett et al. [8] developed an online oral English writing program called the English Oral Presentations, which was mainly used for oral practice. Through the questionnaire survey, the students' feelings after using the program to practice oral English were analysed, and the vast majority of students believed that their oral English level had improved. Dendup and Onthanee [9] found that cooperative learning methods have good effects on students' speaking, reading, and writing skills in English learning. Cooperative learning is an effective teaching strategy to improve English language learning ability. Namaziandost et al. [10] utilized the method of comparative analysis to test the English communication fluency of the two groups of students. They believe that the cooperative learning method can cultivate students' important communication skills in a relatively short period of time. Lin et al. [11] propose a theoretical model of task-based language teaching—interactive revision theory under the theory of second language acquisition. The theory not only emphasizes the importance of language knowledge structure but also expounds the practicality of task-based language teaching. Still, the research has shortcomings. For example, they think that teachers can only correct when students commonly make the same grammatical errors, strengthen correct language forms, and explain the grammar in detail. When students face more complex language structures, they should avoid them while focusing on using vocabulary and communication strategies. The consequence is often to affect the development of the students' interlanguage systems. Sherstinsky [12] divides oral English teaching into three stages, namely pre-task, task loop, and language focus. In the pre-task stage, teachers introduce topics, announce task requirements, and guide learners to carry out activities. The purpose is to assist students in recalling words and phrases that help them complete the task. In the task loop stage, students participate in activities, perform tasks cooperatively, and report the task plan and completion status to the whole class. Finally, the teacher will give corresponding evaluations. In the language focusing stage, the teacher will analyse the language according to the students' written or oral text and explain the language knowledge involved in the task. The advantage of the model is that the language is used in real communication to produce real significance.

In this work, the oral English practice of college students was taken as research content, and deep learning technology was utilized to construct an oral English evaluation model. The performance of the model was analysed by explaining deep learning techniques and the process of English speech recognition. The innovation is that the English oral evaluation model established under the deep learning algorithm had a concordance rate of more than 85% in the recognition of words, speech, and intonation, thereby having a high-quality performance. In addition, it also enriched the relevant theories of oral English teaching to varying degrees and provided a methodological reference for the evaluation of oral English teaching in the future.

## 2. Materials and Methods

### 2.1. Recurrent Neural Network (RNN) System in Deep Learning

**Figure 1:**
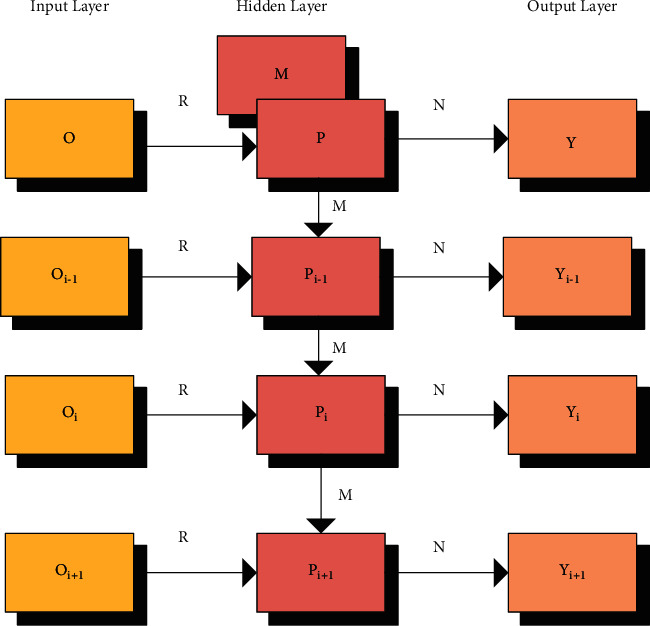
Structural model of RNN.

RNN is a classic model in deep learning and is widely used; together with convolutional neural network (CNN), it has become the basic model of neural networks. CNN has been extremely successful in image processing and has shown its great potential in the ImageNet image recognition competition. AlphaGo is also on the basis of CNN.  Figure 1 shows a widely used RNN structure model.

An RNN is a neural network with memory, which can record the network data information from the starting time to the current moment. That is, the output of the neurons is determined by the data of both current and historical inputs. The processing of data was carried out sequentially, as the input data at the current moment was processed first, followed by the input data at the next moment. As shown in Table 1, there were three optimization features of the RNN.

**Figure 2:**
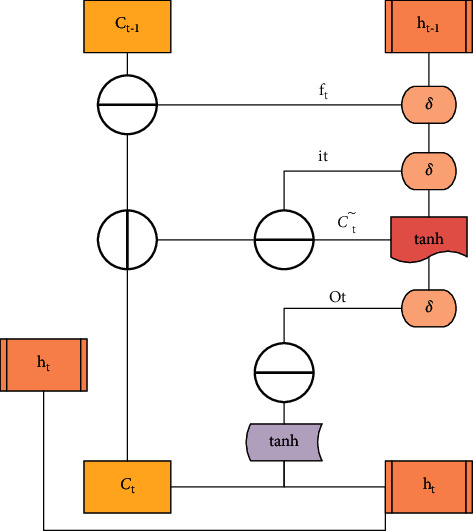
Layout of memory unit in RNN.

The input gate, memory state gate, forget gate, and output gate played different roles in the RNN. Each layer of the deep neural network in Figure 1 was composed of many memory units [13]. The structure of the unit is displayed in Figure 2, consisting of different types of departments and processing functions.

Unlike ordinary neural networks, there was also the forget gate in addition to input gate and output gate. In Figure 2, it represented the input gate, *f*_*t*_ was the forget gate, and *O*_*t*_ was the output gate. *C*^*∼*^_*t*_ was the state of the current input data processed by the tanh function, *C*_*t*_ was the vector value, *δ* was the sign function, and *h*_*t*_ was the output data of the neural unit. These gates could change and extract the data independently and had an influence on the result of the next component, so as to achieve the purpose of autonomous learning and adaptation.

**Figure 3:**
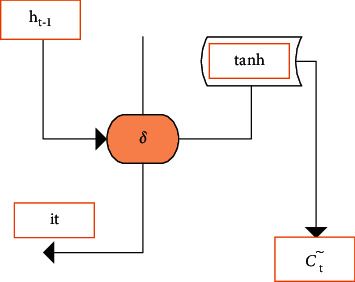
Structure of the input gate.

Figure 3 gives the structure of the input gate and the useful data was updated by filtering the useless information [14].

The flip-flop output *i*_*t*_ and the update output *C*_*t*_^∼^ were solved by the following equations:

(1) $$\begin{array}{c} i_{t}=\delta \left(W_{xi}x_{t}+W_{hi}h_{t-1}+b_{i}\right), \end{array}$$

*C*_*t*−1_ was the output data of the previous neural unit.

(2) $$\begin{array}{c} C_{t}^{∼}=\tanh\left(W_{xc}x_{t}+W_{hc}h_{t-1}+b_{c}\right). \end{array}$$

In the above equations, *W*_*x*_ represented the weight value of the RNN input data, *W*_*h*_ was the weight value of the *t*-th input data in the neuron. *h*_*t*−1_ represented the output data of the previous neuron, and *b* was the corresponding deviation of the neuron; *h*_*t*−1_ was the previous output data of the neural unit. *W*_*xi*_ and *W*_*hi*_ stood for the weight values from the input layer to the corresponding gate, while *W*_*xc*_ and *W*_*hc*_ were the weight values output by the module to the corresponding gate at the previous moment.

**Figure 4:**
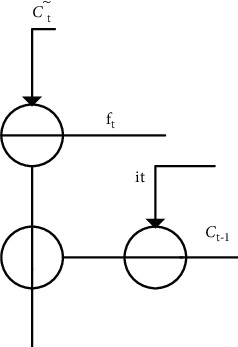
Structure of the memory state gate.

Figure 4 shows the structure of state neural layer, and the trigger output *C*_*t*_ was solved by the following equation:


(3) $$\begin{array}{c} C_{t}=f_{t}C_{t-1}+i_{t}C_{t}^{∼}, \end{array}$$

**Figure 5:**
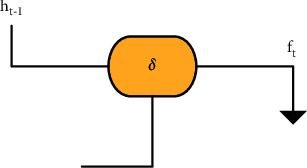
Structure of the forget gate.

The structure of the forget gate is shown in Figure 5, which could save the data stored in the hidden layer selectively according to the sign function [15]. *f*_*t*_ was the trigger output, which was obtained through the following equation:


(4) $$\begin{array}{c} f_{t}=\delta \left(W_{x}x_{t}+W_{hf}h_{t-1}+b_{f}\right). \end{array}$$

**Figure 6:**
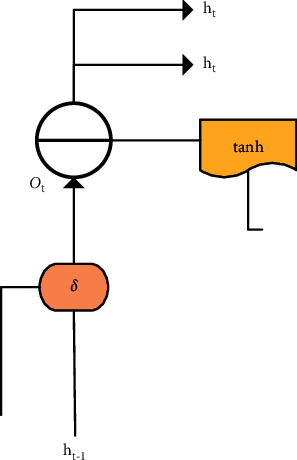
Structure of the output gate.

The output gate could save the current output data of the hidden layer according to the sign function, and control the output of the hidden layer in this way. As its structure is displayed in Figure 6, the trigger output *O*_*t*_ was worked out via the following equation:

(5) $$\begin{array}{c} O_{t}=\delta \left(W_{xo}x_{t}+W_{ho}h_{t-1}+b_{o}\right), \end{array}$$

*W*_*xo*_ and *W*_*ho*_ represent the weight values from the previous neuron to the corresponding gate, and the remaining letters have the same meaning as the above equation. Then, the neural network output *h*_*t*_ could be obtained through the following equation:

(6) $$\begin{array}{c} h_{t}=o_{t}\tanh\left(C_{t}\right). \end{array}$$

The deep neural network was defined as a perceptual node with multiple hidden layers, usually the number of hidden layers was greater than 2. Neurons between adjacent layers were connected by weights. With the input *v*^*l*^, the weight *W*^*l*^, and the bias value *b*^*l*^ of this layer, an activation function could be adopted to obtain the input of the next layer like the following equation:

(7) $$\begin{array}{c} v^{l+1}=f\left(v^{l}W^{l}+b^{l}\right). \end{array}$$

The training method of the deep neural network adopted the error back propagation [16]. The partial derivative of each weight and deviation was obtained through the loss function, to get the descending gradient of the parameters. After that, the gradient descent method was applied for updating the parameters.

The hidden layer of RNN was automatically connected, and the corresponding model could be obtained by extending its hidden layer. *U*, *V*, and *W* were the connection weights of the input layer, output layer, and hidden layer, respectively. The connection weights of all nodes were shared. *x*_*t*_ was the input of the current node, that was, the feature parameter vector obtained from the current speech frame signal. Its hidden layer *s*_*t*_ and output layer *O*_*t*_ are calculated from the following equations:

(8) $$\begin{array}{c} s_{t}=\tanh\left(x_{t}U+s_{t-1}W+b_{s}\right), \end{array}$$

(9) $$\begin{array}{c} O_{t}=s_{t}V. \end{array}$$

The advantage of the self-connection of the hidden layer in RNN was that a network of arbitrary length in time series could be obtained as it was expanded. The speech lengths were also inconsistent. Using the self-connection feature of the hidden layer in RNN, a very flexible input model could be offered for speech signals.

### 2.2. Speech Recognition Method for Oral English Practice

**Figure 7:**
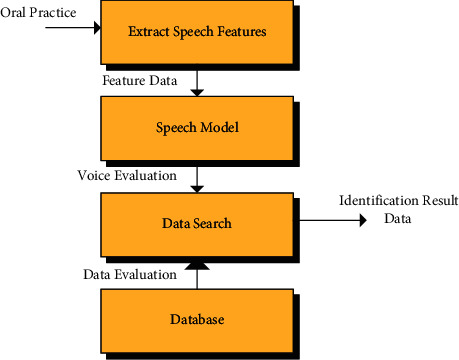
Structure of the speech recognition system.

The speech recognition system was mainly made up of four parts: signal processing and feature extraction, acoustic model (AM), language model (LM), and decoding search [17]. The structure of this system is presented in Figure 7.

The purpose of speech recognition was to allow the machine to understand the natural language spoken by humans. A piece of speech existed in the form of a digital signal in the computer. Obviously, letting the computer understand this digital signal directly would enlarge the calculation of the entire model. As a speech signal was converted into speech features, the dimensions of the parameters could be reduced while ensuring that useful information was preserved to the greatest extent. Moreover, the speech features had better distinguishing characteristics, which was convenient for the modelling of the speech recognition system, with a certain anti-interference ability to the environment.

The speech recognition utilized the sound card of a personal computer (PC) to digitize the speech analog signals and collect the speech signals. According to the Nyquist sampling theorem, in the process of analog/digital signal conversion [18], the sampling frequency f_smax_ was greater than 2 times the highest frequency *F*_max_ in the signal, which could be expressed as the following equation:

(10) $$\begin{array}{c} f_{\text{smax}}\geq 2F_{\text{max}}. \end{array}$$

Afterwards, the sampled digital signal could express the effective information more completely in the original speech signal. Given that the frequency of normal human's speech was generally 40–4000 Hz, the sampling frequency was set to 8 kHz in this work. The obtained speech signals were pre-processed, including pre-emphasis, framing, windowing, and endpoint detection. Then, the feature parameters of the pre-processed speech signal were extracted. Finally, the feature parameters could be selected for model training or pattern matching.

In the pre-emphasis process of oral English speech, the high-frequency end of the average power spectrum of the speech signal was attenuated by 6 dB/octave (oct) above 800 Hz. Therefore, before analysis of the speech signal, a 6 dB/oct high-frequency boosting pre-emphasis digital filter was generally used to boost the high-frequency part of the speech signal [19]. Thereby, the spectrum of the speech signal became flat, which is given in the following equation:

(11) $$\begin{array}{c} H\left(z\right)=1-\alpha^{-1},\;0.9\leq \alpha \leq 1. \end{array}$$

In equation (11), *α* represented the pre-emphasis coefficient, which was usually a constant. Therefore, the pre-emphasized data result *y* (*n*) could be represented by the input speech data *x* (*n*). The specific calculation was expressed as the following equation:

(12) $$\begin{array}{c} y\left(n\right)=x\left(n\right)-\alpha x\left(n-1\right). \end{array}$$

During the framing process, the speech signal was in a state of constant change. Because when a person read English, it could be considered to be basically stable in a relatively short period (10–40 ms) due to the inertial motion of the vocal organs. Thus, the speech signal in this period was called a quasi-steady-state process [20]. The specific calculation is shown in the following equation:

(13) $$\begin{array}{c} \text{Franesper second}=\frac{1}{t}.\left(0.01＜t＜0.03\right). \end{array}$$

In equation (13), *t* was the time period of the data frame, and Franesper second referred to the number of frames of speech per second. For the entire speech data, a frame was a time sequence of parameterized frame features.

In the windowing process, the speech waveform was strengthened near sampling *n* in the speech signal, and the rest of the waveform was weakened. The specific equation is given as follows:

(14) $$\begin{array}{c} Q_{n}=\sum_{m=-\infty }^{\infty }T\left[s\left(n\right)\right]\omega \left(n-m\right). \end{array}$$

In the above equations, *T* [*∗*] represented the linear or nonlinear transformation, *s* (*n*) represented the input speech signal data, and *Q*_*n*_ was the time series of the processed data [21].

**Figure 8:**
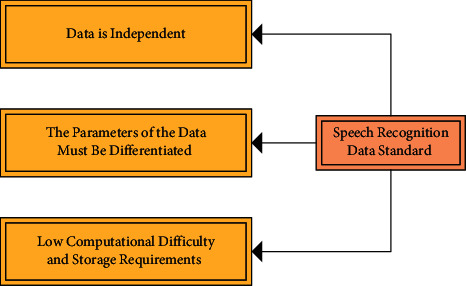
Standards for speech recognition data.

The extraction of speech signal feature parameters was to remove redundant information that was irrelevant to speech processing and to analyse and process speech signals. The original speech signal not only had a very large amount of data but also had too much information that interfered with semantics for different speakers, loudness, length, etc. It was not suitable for direct use in speech processing. The standards for the processing of the speech data are shown in Figure 8.

The quality of the feature parameters would directly affect the performance of speech processing, and a suitable feature extraction method would bring better results. It was necessary to extract feature parameters from the original speech signal. The most ideal speech features described semantic information only, and the total amount of speech data was also small [22].

To apply the grammar error correction algorithm to the actual system, an English grammar error correction system was provided for users. The system architecture and key function design were done in this work. Combined with modern software engineering methods, the system modules were divided, so that the system structure was clear and the function modules were independent. Therefore, the maintainability and expansibility of the system were enhanced.

As the increase in the capacity building requirements of the website was taken into account due to the increase in the number of users, a distributed system architecture of microservices was given. It could cope with the future upgrade and expansion of the system.

The technical parameters in this system were all artificially designed in advance and were definite quantities. These also exist in some fuzzy quantity and grey quantity systems, belonging to uncertainty factors. It could be divided into three categories: cognitive uncertainty, contingent uncertainty, and mixed uncertainty. Cognitive uncertainty, also known as model uncertainty, refers to the uncertainty of model parameters. This uncertainty captures the unknowns of models under collected data, and cognitive uncertainty can be reduced by collecting enough data. Contingent uncertainty refers to the inherent noise in data information that can't be reduced even by collecting more data. Contingent uncertainty can be further divided into homoscedastic uncertainty and heteroscedastic uncertainty. Mixed uncertainty contains both cognitive uncertainty and contingent uncertainty. Due to the limited number of participants in the deep learning scoring model designed in this work, there was mainly cognitive uncertainty. In subsequent studies, the corresponding uncertainties could be mitigated by increasing the number of respondents.

### 2.3. Correction Standards for Speech Recognition of Oral English

**Figure 9:**
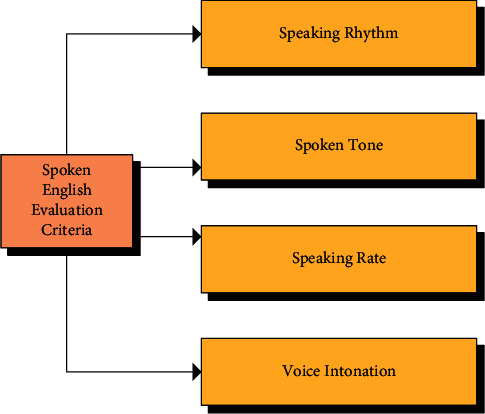
Evaluation standards for oral English.

Subjective evaluation referrs to the evaluation of the pronunciation quality of speech by language experts. The process could generally be divided into three steps. First, after listening to the test speech, the test speech was compared with the standard speech in memory according to the prior knowledge of language accumulated by oneself, and the differences were found at various levels. With the differences, an overall evaluation of the test speech was given. Generally speaking, the pronunciation evaluation results of the test speech by language experts could reflect the pronunciation quality of the test speech and the tester's oral English level more truly. However, because of the differences in knowledge structure and experience among language experts, there might be deviations on the same test speech among different experts. In addition, since the evaluation of speech pronunciation quality is closely related to phonetics and linguistics, but also to physiology and psychology. Even for the same test speech, the evaluation given by the same expert perhaps was different in different states [23]. Thus, the subjective evaluation of pronunciation quality ensured the authenticity of the evaluation results but also exposed the subjective shortcomings. As presented in Figure 9, the evaluation was made mainly from the following four aspects.

Objective evaluation referred to the automatic evaluation by machines of the pronunciation quality of speech. The computer was utilized for objective evaluation of learners' pronunciation quality, which could overcome the shortcomings of subjective evaluation effectively, reduce evaluation deviations, and improve evaluation efficiency. Objective evaluation had unified evaluation standards, which would highlight its advantages when faced with a large number of speech evaluation tasks. The design of the objective evaluation system should simulate the evaluation process of English experts on the test speech as much as possible. Firstly, the feature parameters of the test speech and the standard speech were extracted, respectively. The pattern matching was performed with the trained evaluation model as well as different evaluation indicators, which were compared for the calculation of the machine score of each evaluation indicator. Finally, the machine scores were mapped and fused to obtain the final score of the test speech.

Currently, there are two most common methods for feature analysis of speech evaluation criteria, namely cascade and max pooling. Cascade is all about concatenating and concatenating all feature vectors. If the cascaded fusion is represented by a multiview histogram, one-dimensional column vectors can be obtained, thereby preserving all feature information of each view data [24]. Max pooling is to compute the maximum value of the feature vector and its corresponding position in each view. The calculation is given in the following equation:

(15) $$\begin{array}{c} \hat{r_{i}}=\underset{l\leq i\leq M}{\text{max}}r_{ij}. \end{array}$$

In equation (15), *M* represented the total number of views, and *r*_*ij*_ was the *j*-th feature vector in the *i*-th view.

In the weighted feature fusion on the edge computing side, some image data was lost due to the compression of the model, which would affect the final output result. Thus, the dictionary size in each angle of view was recorded as *N*_*K*_ in advance, then the dictionary size of RNN was *MN*_*K*_, and *M* represented the total number of angles of view. When the angles of view increased, the amount of calculation also increased. In *MN*_*K*_ dictionaries, the data information carried by each word from each angle of view was relatively reduced. Therefore, it was necessary to calibrate the weight of multiview dictionaries, expressed in the following equation:

(16) $$\begin{array}{c} c_{i}=\text{concat}\left(s_{i}^{\left(l\right)},\ldots ,s_{i}^{\left(k\right)},\ldots ,s_{i}^{\left(M\right)}\right),\\ {\text{scale}}_{\text{BoF}}=\text{signmoid}\left(c_{i}\right),\\ \tilde{c_{i}}={\text{scale}}_{\text{BoF}}\cdot c_{i}. \end{array}$$

In the above equations, *c*_*i*_ stood for a set of cascaded histogram vectors of *M* viewing angles, and *s*_*i*_^(*k*)^ represented the viewing angle of the k-th column vector mapping (1 ≤ *K* ≤ *M*). scale_BoF_ was the importance score of each feature data [25].

### 2.4. Experimental Objects and Data Sources

In this work, the English majors at Shenyang Institute of Engineering were tested with the oral English speech recognition model. 20 students were selected in total, including 14 boys and 6 girls. The English sentence patterns were read aloud with the recording system of the model, and 5 sets of sentences were read in total. The sentence patterns are listed as follows:

1. Whatever is worth doing is worth doing well.
2. The hard part isn't making the decision. It's living with it.
3. After all, tomorrow is another day!
4. The driver was drunk and drove the doctor's car directly into the deep ditch.
5. Moonlight city. You just couldn't see an end to it.

After 20 students finished reading the sentence patterns, the results of the recording files were compared using a different speech recognition model with the RNN speech recognition model in this work. It was expected to analyse the performance of the models. The comparative model was the Discrete Hidden Markov Model (DHMM), which was taken as the control group.

## 3. Results and Discussion

### 3.1. Comparison Test of Speech Recognition Rate of Different Models

**Figure 10:**
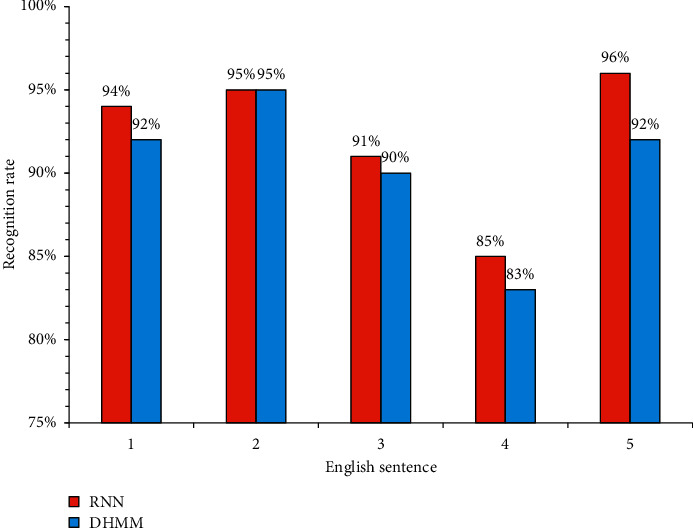
Results of the recognition rate of the systems.

The data used in this work was the test results of the deep learning scoring model. The data were only used for academic research and did not involve personal information about the relevant participants. The above speech recognition model was used as a control. Experiments were then carried out on the constructed RNN speech recognition model. The specific results are presented in Figure 10.

As could be seen from Figure 10, the oral English speech recognition model constructed by RNN in this work had a splendid recognition rate for 5 sets of English sentences, and the lowest recognition rate reached 85%. For the first set of sentence patterns, the recognition rate of the RNN was about 3% higher than that of the DHMM model. For the second set of sentences, the recognition rate of RNN was the same as that of the DHMM model. For the third set of sentences, the recognition rate of RNN was around 1% higher than that of the DHMM model. For the fourth and fifth, the recognition rate of RNN was about 2% and 4% higher than that of the DHMM model, respectively. It was considered that the oral English speech recognition model under the RNN had very good recognition performance. The low recognition rate of students' oral English caused by objective factors could be avoided with great control performance for the evaluation of oral English. In the fourth set, sentences similar to tongue twisters were selected, which resulted in a lower recognition rate than that of other sets of sentence patterns. Therefore, for words with repetitive pronunciation, it was necessary to further improve the recognition performance.

### 3.2. Multifactor Evaluation Results of Oral Reading

**Figure 11:**
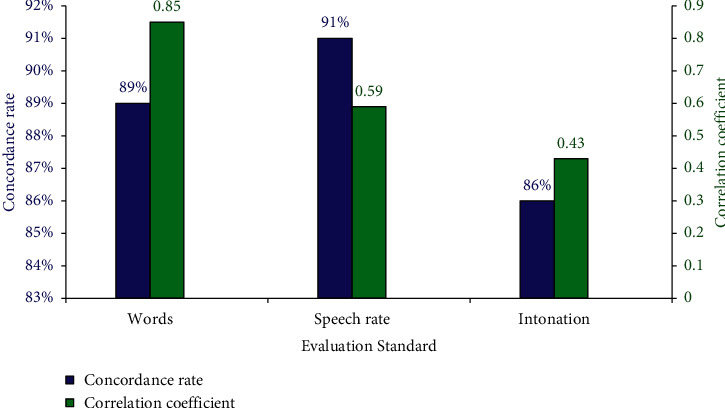
Results of the multifaceted oral English speech recognition.

In the previous analysis above, the recognition rate was compared by using the different speech recognition models from the RNN-based speech recognition model in this work. It was proved that there was a good basis for the recognition performance of the RNN model. Thus, then, the English sentences were tested for words, speech rate, and intonation in oral reading. The correlation coefficient was used as an auxiliary judgment standard to judge whether the evaluation method was feasible. The results are displayed in Figure 11.

In the comprehensive speech analysis of these 5 sets of sentence patterns, it was suggested from the data in Figure 11 that the concordance rate of the speech recognition of RNN was 89% for the accuracy of words, and the correlation coefficient was 0.85. It was greater than 0, indicating that the evaluation method was effective and correct. For the accuracy rate of speech rate, the concordance rate and the correlation coefficient of RNN were 91% and 0.59, respectively, greater than 0, indicating the effective and correct evaluation method. Finally, for the accuracy of intonation, the concordance rate was 86% and the correlation coefficient was 0.43, which was greater than 0 to indicate that the evaluation method was demonstrated to be effective and correct. In general, the oral English speech recognition model under RNN showed excellent evaluation performance, which could be proved with the correlation coefficient. Furthermore, it was also proved that the evaluation methods were accurate and correct, further suggesting that the model could be deployed and applied in the evaluation of oral English.

## 4. Conclusions

As there are many issues in the current oral English teaching evaluation, how to improve the accuracy of the evaluation has become one of the common concerns in education. This work applied the deep learning scoring model to the evaluation of oral English teaching and drew the following conclusions: (1) The deep learning scoring model constructed had a good recognition rate for the selected 5 sets of English sentences, and the lowest recognition rate could reach 85%. (2) The oral English speech recognition model under deep learning showed great evaluation performance, and the correlation coefficients were all greater than 0. This model could be applied to the evaluation of oral English teaching.

Due to limited energy, only some English sentence patterns were selected for this work, and only 20 students participated in the test. The research results had certain particularities. Therefore, the number of research objects and the types of test sentence patterns will be further expanded in follow-up research. The test sentence patterns should include but not be limited to interrogative sentences, rhetorical questions, declarative sentences, compound sentences, and other special sentences patterns to improve the generality of the results.

## Figures and Tables

**Table 1:** RNN optimization features.

Optimization features	Specific optimizations
Dual-directional modelling	The current RNN could only perform calculations under the current information when operating on data information. The dual-directional RNN adopted the ability of bidirectional data infusion and determined the output.

Long-term dependence	When the data sequence was long, the traditional RNN might lead to gradient failure. While the long short-term memory (LSTM) network could solve this issue, with the principle of introducing input gate, forget gate, and output gate.

Optimized computing nodes	Reoptimization was made on the basis of long-term dependence, as remake gate and innovation gate were added. After these two nodes were added, the entire system had a faster computing rate due to reduced parameter scale.

## Data Availability

The data used to support the findings of this study are included within the article.
